# Quantitative computed tomography analysis of changes in airway volume after total laryngectomy

**DOI:** 10.1017/S0022215126104812

**Published:** 2026-06

**Authors:** Melih Akşamoğlu, Nuray Bayar Muluk, Cahit Talha Acar, Orhan Tunç, Teslime Bulut Acar, Mehmet Hamdi Şahan

**Affiliations:** 1 Radiology Department, Faculty of Medicine, Gaziantep University, Gaziantep, Turkiye; 2 ENT Department, Faculty of Medicine, Kırıkkale University, Kırıkkale, Turkiye; 3 ENT Department, Faculty of Medicine, Gaziantep University, Gaziantep, Turkiye

**Keywords:** lung volume measurements, laryngectomy, laryngeal neoplasms

## Abstract

**Objective:**

We evaluated airway volume in patients after total laryngectomy.

**Methods:**

A total of 53 patients who underwent total laryngectomy were included in this study. Twelve of the patients had supraglottic Ca and 41 had transglottic Ca. Total lung volume, and intrapulmonary and tracheal airway volumes were measured.

**Results:**

After total laryngectomy, tracheal airway volume decreased more in transglottic Ca patients compared with supraglottic Ca patients (*p* < 0.05). Even though intrapulmonary airway volumes and total lung volumes were not different between transglottic Ca and supraglottic Ca patients (*p* > 0.05), these volumes decreased post-operatively in both groups (*p* < 0.05). In older patients, the post-operative decrease in the tracheal airway volume was higher than in younger patients (*p* < 0.05). Pre-operative radiotherapy was applied in 50.0 per cent of supraglottic Ca patients and 58.5 per cent of transglottic Ca patients (*p* > 0.05).

**Conclusion:**

Because lower airway volume and total lung volume decrease after total laryngectomy, pulmonary rehabilitation should be administered post-operatively and appropriate follow up is essential to prevent tracheostomal narrowing.

## Introduction

The principal objective in the management of laryngeal malignancies is to achieve effective oncologic control. Additional aims include maintaining functional speech and swallowing, and eliminating the need for a tracheostomy. Therapeutic strategies for laryngeal carcinoma may involve radiotherapy, systemic therapy, surgical intervention or combined approaches. The specific treatment plan is determined by factors such as tumour histology, anatomical site, laryngeal function and the patient’s overall medical and social circumstances. Total laryngectomy remains the standard salvage procedure for recurrent or persistent disease following non-surgical treatments and is also frequently employed as a primary option for individuals with advanced laryngeal tumours.^1^

Effective management often relies on the co-ordinated input of multidisciplinary teams, such as tumour boards or specialised clinics, which guide clinical decision-making. As with other head and neck cancers, key determinants of treatment selection include tumour type, anatomical involvement, laryngeal functionality and patient-specific co-morbidities. Total laryngectomy continues to represent the definitive surgical salvage technique for cases that recur after organ-preservation therapy and is widely utilised as an initial therapy for high-stage disease.^2^

Multiple variables, including anatomic constraints, physiologic status, geographic access to care and occupational demands, can influence therapeutic choices. Among these, the stage of the disease is the most critical factor. Staging depends on the size and spread of the primary lesion and the condition of the regional lymph nodes. Early lesions (stages I and II) are generally amenable to single-modality treatments such as definitive radiotherapy or conservative surgical procedures (e.g. cordectomy or partial laryngectomy). In contrast, advanced tumours (stages III and IV) typically require combined-modality therapy, including chemoradiation or surgical procedures followed by adjuvant radiation.^3^

Because laryngectomy permanently separates the upper and lower airways, the procedure not only eliminates natural voice production but also predisposes patients to long-term pulmonary challenges, such as chronic cough, excessive mucus formation and frequent expectoration. Many survivors also report systemic and quality-of-life difficulties, including fatigue, sleep disturbances, diminished olfactory and gustatory function, and reduced social engagement.^4^

Following total laryngectomy, the upper airway anatomy undergoes a profound alteration that directly affects tracheal airway volume. Potential post-operative narrowing or widening of the airway may have significant effects on respiratory function and patient comfort. The aim of this study was to compare tracheal airway volumes on pre- and post-operative thoracic computed tomography (CT) images in patients who had undergone total laryngectomy. In addition, it was considered that lung volume may also change secondary to the operation. Measurements were performed using images archived in the Gaziantep University Hospital Picture Archiving and Communication System (PACS) system, with the goal of quantitatively demonstrating the anatomical transformation that occurs after a surgical procedure.

## Materials and methods

### Study population and ethical approval

This retrospective study was a collaboration between the Department of Radiology at Gaziantep University Faculty of Medicine and the Department of Otolaryngology at Kırıkkale University Faculty of Medicine. Ethical approval was obtained from the Gaziantep University Non-Interventional Clinical Research Ethics Committee (Decision No: 2025/314, 10.09.2025). All procedures were performed in accordance with the principles of the Declaration of Helsinki.

A total of 53 patients who underwent total laryngectomy between 2014 and 2025, and had both pre- and post-operative thoracic CT images stored in the PACS system were included. The time interval between the pre- and post-operative CT scans was seven months (one month before the operation and six months after the operation).

The age range of the participants was 40–85 years. Twelve patients had supraglottic Ca and 41 had transglottic Ca. Tumour size, subglottic extension and radiotherapy treatment time (none, pre-operative or post-operative) were also noted. The mean smoking status of the patients was 38.39 ± 17.03 pack-years.

In the pre-operative period, all patients were referred for examination with a request for a pulmonology consultation, and the pulmonology department performed pulmonary function tests when deemed necessary. Data from pulmonary function test results are not included in our study dataset. Because no post-operative follow up was recommended for our patients, post-operative pulmonology examinations and pulmonary function tests were not performed. Chronic obstructive pulmonary disease was present in 12 patients (22.2 per cent).

The inclusion criteria were history of total laryngectomy, availability of pre- and post-operative thoracic CT scans and adequate image quality allowing proper segmentation. The exclusion criteria were inadequate image quality, missing post-operative CT, patients who underwent revision surgery in the post-operative period, technical inadequacy preventing segmentation, the presence of new post-operative pathologies (e.g. lung metastasis, obstructive lung disease, pneumonia) and the presence of a massive pleural effusion affecting the measurements.

### Patient selection process

**Figure 1.**
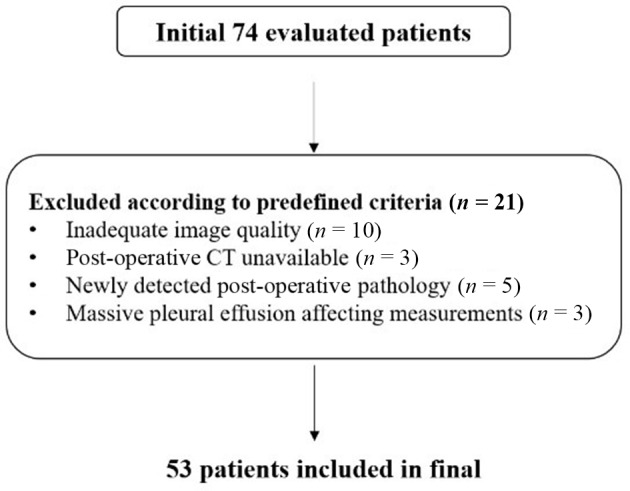
Patient selection flow diagram. CT = computed tomography. Figure 1 long description.

The study population was formed according to the criteria outlined above, with 74 initially identified patients. Patients were excluded for four reasons: inadequate image quality (10 patients), missing post-operative CT (3 patients), new post-operative pathology (5 patients) and massive pleural effusion affecting measurements (3 patients). A total of 53 patients were included in the final analysis. A flow diagram of the patient selection process is shown in Figure 1.

### Sample size power analysis

A power analysis was performed using G*Power 3.1. With effect size *d* = 0.5, *α* = 0.05 and power (1 − *β*) = 0.80, the minimum required sample size was determined to be 34 participants. The final sample of 53 patients exceeded this threshold.

### Methodological rationale: determination of anatomical and radiological boundaries

When determining the upper and lower boundaries for airway volume measurement on pre- and post-operative CT images, surgical anatomy, radiological segmentation principles and relevant literature were considered.^5^^,^^6^

### Computed tomography scan protocols and measurements

All scans were acquired through standard thorax CT imaging. All chest CT examinations were performed using a 160-slice multidetector computed tomography scanner (Canon Aquilion Prime SP; Canon Medical Systems, Otawara, Japan) at 120 kVp with automatic exposure control. The scans were obtained during full-inspiration breath-hold and with intravenous contrast enhancement. The examinations were conducted with a slice thickness of 5 mm, a field of view adjusted between 400 and 450 mm depending on the patient, and an image matrix of 512 × 512. The images were transferred to a commercially available Vitrea workstation (Vital Images, Minnetonka, MN, USA), and the tracheal, airway and lung volume measurements were performed on the Vitrea workstation using the pulmonary analysis module (version 7.12.3.133).

All measurements were determined by consensus between two radiologists and subsequently performed by the same observer (MA and CTA).

### Lung volume measurement (total lung volume)

**Figure 2.**
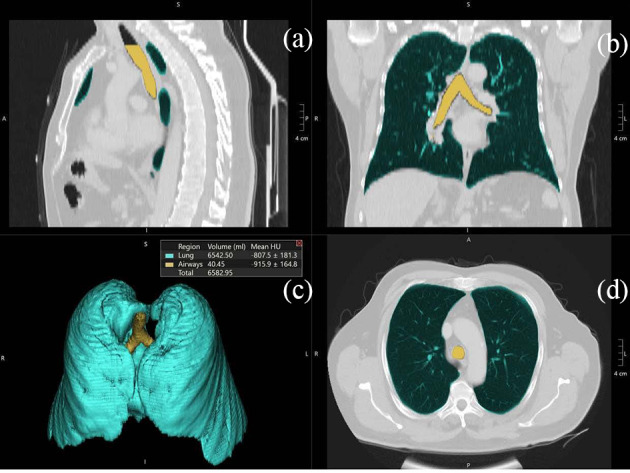
CT-based volumetric assessment of total lung parenchyma and airway volumes in sagittal (A), coronal (B), three-dimensional volumetric reconstruction images demonstrating calculated total lung parenchymal and airway volumes (C), and axial (D) images. Figure 2 long description.

The pulmonary analysis software was used to obtain lung volume. Total lung and airway measurements were automatically calculated by the program, and under- or over-segmented areas were manually corrected. The combined volume of both lungs was accepted as the total lung volume (Figure 2).

### Airway volume measurement (intrapulmonary airway volume)

**Figure 3.**
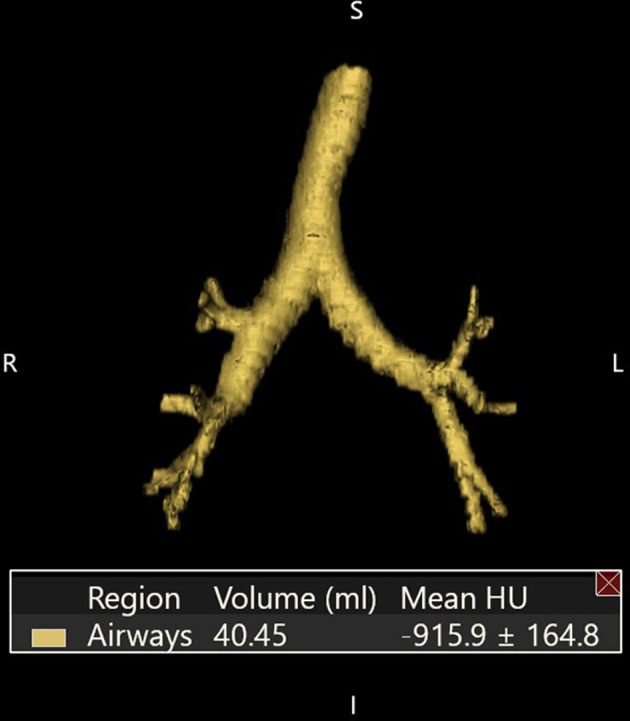
Three-dimensional visualisation of the airways and airway volume measurement. Figure 3 long description.

On axial images, the first slice in which the superior margin of the sternum became visible (jugular notch level) was defined as the mediastinal entry level. Airway volume was calculated starting from this level and included the trachea, main bronchi, lobar bronchi and segmental bronchi bilaterally, while subsegmental branches were excluded (Figure 3).^7^

### Tracheal volume measurement (tracheal airway volume)

**Figure 4.**
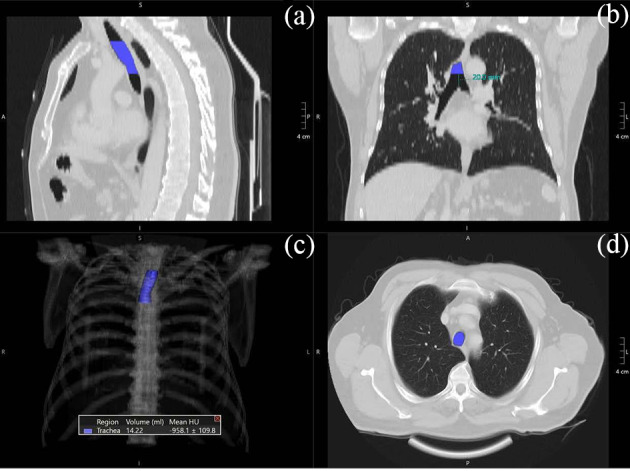
Tracheal volume measurement shown in sagittal (A), coronal (B), three-dimensional volumetric reconstruction with calculated volume (C), and axial (D) images. The measured segment extends from the jugular notch superiorly to 2 cm above the carina inferiorly. . Figure 4 long description.

For tracheal volume, the segment extending from the mediastinal entry level to 2 cm superior to the carina was measured (Figure 4).

### Rationale for boundary selection

The jugular notch was chosen as the upper boundary because it represents the anatomically stable starting point of the intrathoracic trachea.

The level 2 cm superior to the carina was selected to avoid respiratory motion and geometric variation associated with the carinal bifurcation.

This interval represents the most stable segment used in previous studies analysing the intrathoracic trachea and ensures high reproducibility in volume measurement. Prior studies have used 1–3 cm above the carina as the lower boundary,^5^^,^^6^ therefore we selected 2 cm for consistency with this range.

This methodological framework aligns with previous studies assessing intrathoracic airway geometry,^5^^,^^6^ lung aeration^8^ and airway segmentation optimisation.^7^

### Statistical analysis

The data obtained in the study were analysed using SPSS for Windows 26.0 software (SPSS, Inc., IBM, Chicago, IL, USA). The Mann–Whitney U test, Wilcoxon signed rank test, chi-square (*χ*^2^) test and Spearman’s correlation rho efficient test were used.

Related to the difference in the number of patients between the supraglottic (*n* = 12) and transglottic (*n* = 41) groups, non-parametric statistical tests were used for the statistical analysis. A power analysis was performed using G*Power 3.1. A value of *p* less than 0.05 was considered statistically significant.

## Results

There were 11 males (91.7 per cent) and 1 female (8.3 per cent) in the supraglottic Ca group and 36 males (87.8 per cent) and 5 females (12.2 per cent) in the transglottic Ca group (*p* = 1.000, *χ*^2^ = 0.000). The mean age of the supraglottic Ca group was 67.83 ± 11.55 years and in the transglottic Ca group it was 66.46 ± 9.95 years (*p* > 0.05) (Table 1).

**Table 1.** Measurement results in supraglottic Ca and transglottic Ca groups Table 1 long description.

Parameter	Group 1 (supraglottic Ca) (*n* = 12	Group 2 (transglottic Ca) (*n* = 41)	p*
Age (mean ± SD (median); years)	67.83 ± 11.55 (69.00)	66.46 ± 9.95 (67.00)	0.558
Tracheal airway volume (mean ± SD (median); ml)			
– Pre-operative	18.99 ± 6.65 (16.75)	14.82 ± 3.48 (14.47)	0.054
– Post-operative	16.66 ± 5.57 (15.29)	12.57 ± 3.30 (12.20)	0.014
– Volume change	2.32 ± 1.32 (2.04)	2.24 ± 0.93 (2.19)	0.758
– *p*^preop–postop^**	0.002	0.000	
Intrapulmonary airway volume (mean ± SD (median); ml)			
– Pre-operative	37.04 ± 9.22 (34.62)	32.65 ± 7.99 (32.63)	0.167
– Post-operative	31.71 ± 9.57 (30.67)	28.27 ± 7.32 (27.90)	0.350
– Volume change	5.32 ± 2.08 (5.37)	4.38 ±1.64 (4.14)	0.158
– *p*^preop–postop^**	0.002	0.000	
Total lung volume (mean ± SD (median); ml)			
– Pre-operative	5800.75 ± 1488.34 (5051.35)	5114.94 ± 1344.23 (5150.08)	0.298
– Post-operative	5051.90 ± 1349.06 (4716.43)	4372.35 ± 1364.70 (4321.20)	0.177
– Volume change	748.84 ± 516.18 (786.60)	742.59 ± 534.12 (652.69)	0.782
– *p*^preop–postop^**	0.004	0.000	
Tumour diameter (*n* (%))			p***
–< 2 cm	4 (33.3)	2 (4.9)	*p* = 0.045
– 2−4 cm	6 (50.0)	30 (73.1)	*χ*^2^ = 6.191
– >4 cm	2 (16.7)	9 (22.0)	
Subglottic extension (*n* (%))			
– <5 mm	12 (100.0)	12 (29.3)	*p* = 0.000
– 5−10 mm	0 (0.0)	12 (29.3)	*χ*^2^ = 23.429
– >1 cm	0 (0.0)	17 (41.5)	
Radiation therapy (RT) (*n* (%))			
– None	6 (50.0)	16 (39.0)	*p* = 0.442
– Pre-operative	6 (50.0)	24 (58.5)	*χ*^2^ = 0.592
– Post-operative	0 (0.0)	1 (2.5)	

* *p* value shows the results of Mann–Whitney U test; **p^preop–postop^ value shows the results of the Wilcoxon signed ranks test; ***p value shows the results of the chi-square (*χ*^2^) test.

### Tumour diameter

In the transglottic Ca group, most of the tumour diameters were 2–4 cm or more than 4 cm. However, in the supraglottic Ca group, tumour diameters were 2 or 2–4 cm (*p* = 0.045, *χ*^2^ = 6.191) (Table 1).

### Subglottic extension

In the supraglottic Ca group, subglottic extension was less than 5 mm in all patients, but in the transglottic Ca group, subglottic extension was 5–10 mm in 41.5 per cent of patients (*p* = 0.000, *χ*^2^ = 23.429).

### Radiotherapy

In both groups, pre-operative radiotherapy was given to patients, 50.0 per cent in the supraglottic Ca group and 58.5 per cent in the transglottic Ca group (*p* > 0.05).

### Tracheal airway volume

The tracheal airway volume of the transglottic Ca group (12.57 ± 3.30 ml) was significantly lower than that of the supraglottic Ca group (16.66 ± 5.57 ml) (*p* < 0.05) (Table 1). In each of the groups, tracheal airway volumes decreased in the post-operative period compared with the pre-operative period (*p* < 0.05)

### Intrapulmonary airway volume

There were no significant differences between intrapulmonary airway volumes of the supraglottic Ca and transglottic Ca groups in the pre- and post-operative periods (*p* > 0.05). In each of the groups, intrapulmonary airway volumes decreased in the post-operative period compared with the pre-operative period (*p* < 0.05).

### Total lung volume

There were no significant differences between the total lung volumes of the supraglottic Ca and transglottic Ca groups in the pre- and post-operative periods (*p* > 0.05). In each of the groups, total lung volumes decreased in the post-operative period compared with the pre-operative period (*p* < 0.05)

The correlation test results are shown in Table 2. There were positive correlations between tracheal airway volumes, intrapulmonary airway volumes and total lung volumes in the pre- and post-operative periods (*p* < 0.05).

**Table 2.** Correlation test results Table 2 long description.

		Tracheal airway volume (ml)			Intrapulmonary airway volume (ml)			Total lung volume (ml)		
		Pre-operative	Post-operative	Volume change	Pre-operative	Post-operative	Volume change	Pre-operative	Post-operative	Volume change
Tracheal airway volume (ml)										
– Pre-operative	*r*		0.960	0.374	0.769	0.793	0.202	0.509	0.424	0.263
	p*		**0.000**	**0.006**	**0.000**	**0.000**	0.147	**0.000**	**0.002**	0.057
– Post-operative	*r*	0.960		0.15	0.726	0.753	0.160	0.516	0.461	0.186
	p*	**0.000**		0.279	**0.000**	**0.000**	0.254	**0.000**	**0.001**	0.181
– Volume change	*r*	0.374	0.151		0.312	0.313	0.127	0.112	0.003	0.304
	p*	**0.006**	0.279		**0.023**	**0.022**	0.363	0.425	0.981	**0.027**
Intrapulmonary airway volume (ml)										
– Pre-operative	*r*	0.769	0.726	0.312		0.965	0.337	0.638	0.577	0.149
	p*	**0.000**	**0.000**	**0.023**		**0.000**	**0.014**	**0.000**	**0.000**	0.286
– Post-operative	*r*	0.793	0.753	0.313	0.965		0.124	0.642	0.564	0.169
	p*	**0.000**	**0.000**	**0.022**	**0.000**		0.378	**0.000**	**0.000**	0.228
– Volume change	*r*	0.202	0.160	0.127	0.337	0.124		0.113	0.179	0.039
	p*	0.147	0.254	0.363	**0.014**	0.378		0.419	0.200	0.781
Total lung volume (ml)										
– Pre-operative	*r*	0.509	0.516	0.112	0.638	0.642	0.113		0.897	0.189
	p*	**0.000**	**0.000**	0.425	**0.000**	**0.000**	0.419		**0.000**	0.176
– Post-operative	*r*	0.424	0.461	0.003	0.577	0.564	0.179	0.897		−0.197
	p*	**0.002**	**0.001**	0.981	**0.000**	**0.000**	0.200	**0.000**		0.158
– Volume change	r	0.263	0.186	0.304	0.149	0.169	0.039	0.189	−0.197	
Tumour localisation (Code 1: Supralottic Ca, Code 2: Transglottic Ca)	*r*	−0.267	−0.342	0.043	−0.192	−0.130	−0.196	−0.144	−0.187	−0.038
	p*	0.054	**0.012**	0.761	0.169	0.355	0.160	0.302	0.180	0.785
Tumour diameter (Code 1: <2 cm, Code 2: 2−4 cm, Code 3: >4 cm)	*r*	0.019	0.002	−0.063	−0.048	−0.070	0.197	−0.104	−0.194	0.204
	p*	0.894	0.989	0.656	0.730	0.618	0.158	0.459	0.165	0.142
Subglottic extension (Code 1: <5 mm, Code 2: 5−10 mm, Code 3: 5−10 mm)	*r*	−0.146	−0.198	0.150	−0.140	−0.127	0.006	−0.011	−0.083	0.140
	p*	0.298	0.155	0.282	0.316	0.366	0.964	0.937	0.554	0.318
Age (years)	*r*	0.158	0.114	0.296	−0.060	−0.011	−0.177	−0.036	−0.093	0.129
	p*	0.260	0.417	**0.031**	0.671	0.940	0.205	0.798	0.506	0.355
Gender (Code 1: Male, Code 2: Female)	*r*	−0.047	−0.078	0.019	−0.175	−0.125	−0.214	−0.202	−0.253	0.019
	p*	0.740	0.579	0.890	0.210	0.374	0.124	0.146	0.068	0.890

* *p* value shows the results of Spearman’s correlation rho efficient test; *r* shows correlation coefficient; Bold values show that p values were less than 0.05 and statistically significant.

In the transglottic Ca group, post-operative tracheal airway volume decreased compared with the supraglottic Ca group (*p* < 0.05). In older patients, the volume change (post-operative decrease) of the tracheal airway volume was higher than in younger patients (*p* < 0.05). There were no significant differences between the airway volumes of male and female patients (*p* > 0.05).

## Discussion

Total laryngectomy produces substantial alterations in respiratory physiology. Removal of the larynx and creation of a permanent tracheostoma fundamentally modify airway dynamics. The separation of the upper and lower airways reduces the normal pressure gradient between the alveoli and the trachea, and displaces the equal-pressure point towards smaller, more peripheral bronchi. These shifts impair intrapulmonary gas exchange.^9^^,^^10^ In addition, inhaled air no longer undergoes the usual warming, humidification and filtration provided by the nasal and pharyngeal passages.^9^^–^^14^ As a consequence, the tracheobronchial mucosa often becomes hyperreactive, resulting in increased secretion production, crust formation and persistent coughing. These chronic symptoms are frequently associated with measurable declines in pulmonary performance.^15^^–^^17^ Histopathological studies have further demonstrated notable structural changes following laryngectomy, including squamous metaplasia of the ciliated respiratory epithelium and chronic inflammation of the tracheal lamina propria near the carina.^18^^,^^19^

Following the procedure, the entire trachea is redirected to an anterior cervical stoma, while the residual pharynx is reconstructed into a neopharynx, leading to complete anatomical separation of the breathing and swallowing pathways. Consequently, nasal or oral ventilation is no longer possible because both openings now lead solely to the oesophagus. All respiration, along with any oxygen therapy or airway instrumentation, must occur through the neck stoma, which offers minimal natural protection for the lower airway. Although aspiration through the mouth or nose is eliminated, patients must remain cautious around water and potential foreign bodies, which can enter the airway directly via the exposed stoma.^20^

Despite these significant physiologic alterations, the assessment of pulmonary function in laryngectomised individuals has received limited scientific attention, largely because of technical challenges in performing standard tests rather than a lack of clinical necessity.^21^^,^^22^

This study was a retrospective observational investigation aimed at comparing tracheal airway and lung volumes measured pre- and post-operatively in patients who underwent total laryngectomy. In the present study, we evaluated airway volume after total laryngectomy in patients with supraglottic or transglottic Ca through standard neck and thorax CT imaging. In the transglottic Ca group, most of the tumour diameters were 2–4 cm or more than 4 cm. However, in the supraglottic Ca group, tumour diameters were 2 cm or 2–4 cm (*p* < 0.05). In the supraglottic Ca group, subglottic extension was less than 5 mm in all patients, but in the transglottic Ca group, subglottic extension was 5–10 mm in 41.5 per cent of patients (*p* < 0.05). In both groups, pre-operative radiotherapy was given to patients, 50.0 per cent in the supraglottic Ca group and 58.5 per cent in the transglottic Ca group (*p* > 0.05).

The tracheal airway volume of the transglottic Ca group (12.57 ± 3.30 ml) was significantly lower than that of the supraglottic Ca group (16.66 ± 5.57 ml). There were no significant differences between intrapulmonary airway volumes and total lung volumes between groups. In each of the groups, tracheal airway volumes, intrapulmonary airway volumes and total lung volumes decreased in the post-operative period compared with the pre-operative period. It is thought that fibrotic healing, stenosis, scar formation and possible deformities that develop in the tracheal segment after removal of the larynx may reduce airway diameter and consequently its volume.

Because many laryngectomised individuals have a significant smoking history and subsequently breathe under non-physiological airway conditions, multiple alterations in pulmonary performance are observed after surgical procedures. Air drawn directly through the tracheostoma bypasses the upper respiratory tract, eliminating its role in filtering airborne particulates and aerosols. Moreover, the inspired air no longer undergoes humidification or warming. Compared with airflow through the natural upper airway, respiration via a tracheostoma substantially lowers aerodynamic resistance during both inhalation and exhalation, a change that can adversely influence ventilation in the peripheral lung regions.^23^^–^^25^ Progressive impairment of pulmonary function represents one of the key prognostic indicators for survival among individuals who have undergone total laryngectomy.^25^^,^^26^

Accurate assessment of lung function is therefore valuable in this population, not only for mitigating peri-operative risks during subsequent procedures, but also for evaluating treatment outcomes and identifying early functional decline.^27^ Studies have shown that airway resistance increases following laryngectomy, reaching its peak approximately 6 months post-operatively—at nearly 10 times the pre-operative level—and later declining to roughly double the baseline value by 1 year.^28^

A further contributor to reduced tracheal airway volume in patients with transglottic carcinoma is tracheomalacia. This disorder is characterised by excessive collapsibility of the tracheal wall and reduced luminal diameter during the expiratory phase. When the bronchi are also involved, the condition is termed tracheobronchomalacia. Secondary or acquired forms are relatively frequent in individuals with prolonged or repeated endotracheal intubations or underlying chronic obstructive pulmonary disease.^29^^,^^30^ Structural weakening can arise from atrophy or loss of the tracheal elastic fibre framework or from fragmentation of the cartilaginous rings.^31^ Such changes promote marked expiratory airway collapse, typically defined as a greater than 50 per cent reduction in tracheal cross-sectional area relative to inspiration.^32^

In our study, there were positive correlations between tracheal airway volumes, intrapulmonary airway volumes and total lung volumes in the pre- and post-operative periods. In the transglottic Ca group, post-operative tracheal airway volume decreased compared with the supraglottic Ca group. In older patients, the change (post-operative decrease) in the tracheal airway volume was higher than in younger patients.

Ageing is associated with progressive structural and functional changes in the respiratory system, particularly affecting airway elasticity and overall pulmonary performance. Elastic recoil of the lungs decreases as a result of alterations in elastin and collagen fibres, leading to increased lung compliance but reduced expiratory flow rates. At the same time, chest wall stiffness increases and respiratory muscle strength declines, contributing to reduced ventilatory efficiency.^33^^–^^35^

Multiple investigations indicate that a considerable proportion of laryngectomised individuals may benefit from pharmacologic management because of the high prevalence of airflow obstruction in this population.^25^^,^^26^^,^^36^ Using the Medical Research Council Dyspnea Scale,^37^ more than half of patients reported shortness of breath only during vigorous physical activity, while approximately one-third experienced dyspnoea when walking briskly or ascending mild inclines. These findings may be partly attributable to smoking cessation following surgery because improvements in respiratory symptoms can occur relatively soon after quitting.^38^

According to Castro *et al*.^21^ the post-laryngectomy condition is marked by notable impairments in respiratory function. In their study of 50 patients who had undergone total laryngectomy at least 6 months earlier, 56 per cent exhibited abnormal breathing patterns. Among these, 14 patients showed an obstructive pattern without air trapping, 11 demonstrated obstruction with air trapping and only 3 presented a restrictive pattern. On average, a reduction in diffusing capacity (74.3 per cent of patients) and an increase in airway resistance (121.7 per cent of patients) were observed.

In the study by Durán Cantolla *et al*.^39^ among 30 individuals assessed after laryngectomy, 47 per cent demonstrated ‘normal pulmonary function’, whereas 53 per cent showed some degree of impairment. Vázquez de la Iglesia *et al*.^26^ reported an even higher prevalence of obstructive ventilatory patterns, identifying such changes in 81 per cent of their cohort. Ackerstaff *et al*.^24^ suggested that the pronounced respiratory dysfunction frequently observed in these patients may stem from the increased susceptibility to airway infections associated with the tracheostoma. Additionally, many laryngectomy patients enter surgery with compromised pulmonary reserve as a result of long-standing tobacco use, placing them at heightened risk for rapid respiratory decline.^40^ Consequently, timely identification of respiratory decompensation and prompt therapeutic intervention are essential.^41^

Growing recognition of the adverse pulmonary consequences of total laryngectomy has motivated efforts to explore rehabilitation strategies aimed at improving respiratory outcomes in these patients.^42^^–^^45^ Although several studies have examined long-term pulmonary changes following the procedure,^17^ research addressing its immediate effects remains limited.^11^^–^^14^

In our study, pre-operative radiotherapy was given to patients, 50.0 per cent in the supraglottic Ca group and 58.5 per cent in the transglottic Ca group. Radiotherapy triggers fibrosis, known as radiation-induced fibrosis, a process characterised by excessive synthesis and disorganised deposition of extracellular matrix components, particularly collagen, as part of an abnormal wound-healing response. In individuals treated for head and neck malignancies, this fibrotic transformation represents a frequent and, in many cases, unavoidable late effect as a result of repeated radiation-induced tissue injury.^46^ The development of radiation-induced fibrosis can be understood as a multistep pathological process involving three overlapping phases. Initially, during the inflammatory phase, radiation causes cellular and tissue damage, which stimulates the release of pro-inflammatory cytokines. This is followed by a proliferative phase, marked by activation of fibroblasts and their differentiation into myofibroblasts. In the final remodelling phase, disruption of extracellular matrix regulation leads to its progressive accumulation, ultimately resulting in fibrotic tissue changes.^47^

### Limitations

The retrospective design of the study and the use of thicker CT slices introduced certain limitations in volumetric assessment. The need for occasional manual correction of the automated segmentation increased operator dependency and limited the reproducibility of the measurements.

Data from pulmonary function test results are not included in our study dataset, which is another limitation of our study.

- After total laryngectomy, tracheal airway volume decreased more in transglottic Ca compared with supraglottic Ca
- Even though intrapulmonary airway volumes and total lung volumes were not different between transglottic Ca and supraglottic Ca, these volumes decreased post-operatively in both groups
- There were positive correlations between tracheal airway volumes, intrapulmonary airway volumes and total lung volumes in pre- and post-operative periods
- In older patients, volume change (post-operative decrease) of the tracheal airway volume is higher than in younger patients
- Because lower airway volume and total lung volume decrease after total laryngectomy, pulmonary rehabilitation should be administered post-operatively

## Conclusion

After total laryngectomy, tracheal airway volume decreased more in transglottic Ca patients compared with supraglottic Ca patients. Even though intrapulmonary airway volumes and total lung volumes were not different between transglottic Ca and supraglottic Ca patients, these volumes decreased after the post-operative period in both groups of patients. Because lower airway volume and total lung volume decrease after total laryngectomy, pulmonary rehabilitation should be administered post-operatively, and appropriate follow up is essential to prevent tracheostomal narrowing.

## Figure 1 Long description

A flowchart illustrating the patient selection process. It begins with 'Initial 74 evaluated patients' at the top. Below, a box lists exclusion criteria: inadequate image quality (10 patients), post-operative CT unavailable (3 patients), newly detected post-operative pathology (5 patients) and massive pleural effusion affecting measurements (3 patients), totaling 21 exclusions. The process concludes with '53 patients included in final' at the bottom.

Navigate back to Figure 1.

## Figure 2 Long description

The image A shows a sagittal view of the lungs with highlighted airways. The image B shows a coronal view of the lungs with airways marked. The image C shows a three-dimensional reconstruction of the lungs, displaying the airway structure. The image D shows an axial view of the lungs with the airways highlighted. Each view provides a different perspective for assessing lung and airway structures.

Navigate back to Figure 2.

## Figure 3 Long description

A three-dimensional visualization of the airways is shown, with a branching structure resembling the trachea and bronchi. The image includes a data box displaying 'Region: Airways', 'Volume: 40.45 milliliters' and 'Mean HU: negative 915.9 plus minus 164.8'. The letters S, R, L and I are positioned around the image, likely indicating anatomical directions.

Navigate back to Figure 3.

## Figure 4 Long description

The image A shows a sagittal plane view of the trachea with highlighted measurement. The image B shows a coronal plane view of the trachea with measurement indicated. The image C shows a three-dimensional reconstruction of the trachea with volumetric calculation displayed. The image D shows an axial plane view of the trachea with measurement highlighted.

Navigate back to Figure 4.

## Table 1 Long description

The table compares measurement results between supraglottic and transglottic cancer groups. Supraglottic patients have higher pre-operative tracheal airway volumes (18.99 ml) than transglottic patients (14.82 ml). Post-operative reductions are significant in both groups, with supraglottic patients showing a larger decrease in total lung volume. Tumor diameters are generally smaller in the supraglottic group, with 33.3% having tumors of 2 cm compared to 4.9% in the transglottic group. Subglottic extension is less than 5 mm in all supraglottic cases, while transglottic cases show more variability. Radiotherapy is more common pre-operatively in the transglottic group. Statistical tests indicate significant differences in several parameters.

Navigate back to Table 1.

## Table 2 Long description

The table presents correlation coefficients and p-values for pre-operative, post-operative, and volume change measurements of tracheal, intrapulmonary, and total lung volumes. Notably, pre-operative tracheal airway volume shows a strong positive correlation with post-operative total lung volume (r=0.897, p<0.001), suggesting significant post-surgical changes. Intrapulmonary airway volume pre-operative and post-operative values also exhibit high correlation (r=0.965, p<0.001). Tumor localization and diameter show weaker correlations with airway and lung volumes, with p-values often above 0.05, indicating less statistical significance. Age and gender have minimal impact on volume changes, as reflected by low correlation coefficients and high p-values. These findings highlight the importance of airway volume measurements in assessing surgical outcomes.

Navigate back to Table 2.
